# Synthetic Potential of Regio- and Stereoselective Ring Expansion Reactions of Six-Membered Carbo- and Heterocyclic Ring Systems: A Review

**DOI:** 10.3390/ijms24076692

**Published:** 2023-04-03

**Authors:** Rida Noor, Ameer Fawad Zahoor, Asim Mansha, Samreen Gul Khan, Atta Ul Haq, Sajjad Ahmad, Sami A. Al-Hussain, Ali Irfan, Magdi E. A. Zaki

**Affiliations:** 1 Department of Chemistry, Government College, University Faisalabad, Faisalabad 38000, Pakistan; ridanoor5275@gmail.com (R.N.); asimmansha@gcuf.edu.pk (A.M.); samreengul@gcuf.edu.pk (S.G.K.); attaulhaq@gcuf.edu.pk (A.U.H.); raialiirfan@gmail.com (A.I.); 2 Department of Chemistry, University of Engineering and Technology Lahore, Faisalabad Campus, Faisalabad 38000, Pakistan; sajjad.ahmad@uet.edu.pk; 3 Department of Chemistry, College of Science, Imam Mohammad Ibn Saud Islamic University (IMSIU), Riyadh 11623, Saudi Arabia; sahussain@imamu.edu.sa

**Keywords:** six-member rings, heterocyclic ring expansion, azepine, azulene, biological activities

## Abstract

Ring expansion reactions fascinate synthetic chemists owing to their importance in synthesizing biologically active compounds and their efficacy in medicinal chemistry. The present review summarizes a number of synthetic methodologies, including stereoselective and regioselective pathways adopted by scientists, for framing medium- to large-size carbo- and heterocycles involving lactams, lactone, azepine and azulene derivatives via ring expansion of six-membered carbo- and heterocycles that have been reported from 2007–2022. Numerous rearrangement and cycloaddition reactions involving Tiffeneau–Demjanov rearrangement, Aza–Claisen rearrangement, Schmidt rearrangement, Beckmann rearrangement, etc., have been described in this regard.

## 1. Introduction

**Figure 1:**
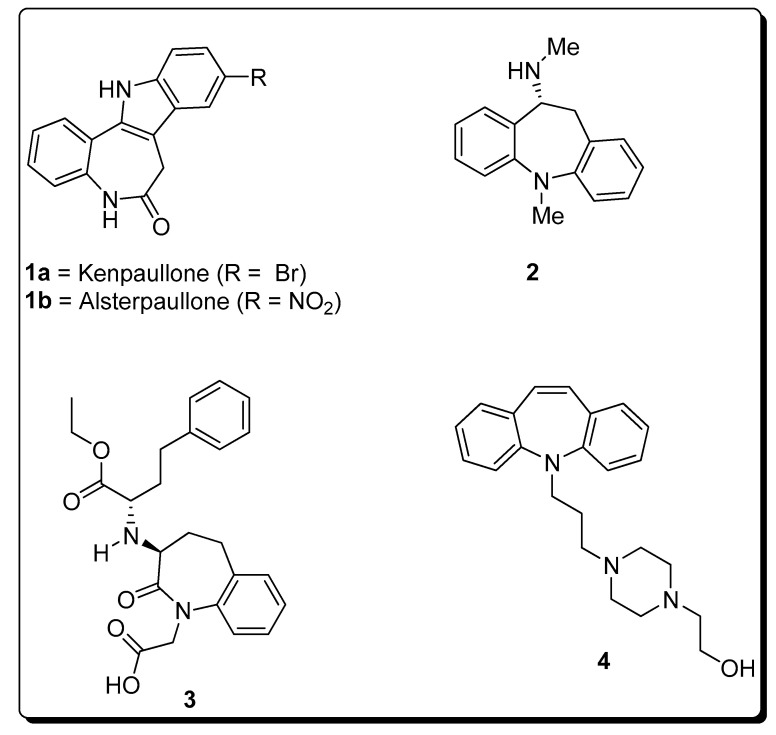
Structures of kenpaullone, alsterpaullone (**1a,b**), metapramine **2**, benazepril **3** and opipramol **4**.

Ring expansion reactions are among the most efficient reactions in chemistry that facilitate the synthesis of functionalized medium- to large-size ring systems. During recent years, efforts have been put forward by the scientists towards the synthesis of large-size carbo- as well as heterocycles via ring expansion reactions. Six-membered ring expansion reactions are of great importance for synthesizing 7- to 12- and higher-membered rings. The large-size carbo- and heterocyclic rings are of great interest for organic chemists due to their medicinal properties [1]. Benazepril **3** [2] is an angiotensin-converting enzyme inhibitor that has beneficial effects in the medication of diabetic kidney disease, hypertension and heart failure [3]. Paullones [4] are derived from indole [3,2-d]-1-benzoazepin-2-one, which prevents cyclin-dependent kinases (CDKs). Kenpaullone and alsterpaullone (**1a,b**) [5] have demonstrated the anticancer activity and CDK inhibitory activity [6,7]. Metapramine **2** [8] and opipramol **4** [9] are used to cure depression (Figure 1). Some of the large-size carbocyclic/heterocyclic compounds exhibit various biological activities, such as antitumor activity [10], inhibition of HIV-1 replication [11], antinociception activity [12], antiepileptic activity [13], inhibition of *α*-glucosidase [14] and inhibition of cytidine deaminase.

Herein, we report the recent synthetic approaches used for the formation of large-size ring lactams, lactones, O, N, S-containing heterocyclic rings, substituted azulenes and azepine derivatives. A number of rearrangements, including Aza–Claisen rearrangement [15], Beckmann rearrangement [16], Tiffeneau–Demjanov rearrangement [17], Schmidt rearrangement and cycloaddition reactions (10 + 4) [18] and (6 + 6) [19], are discussed in the current review.

## 2. Literature Review

### 2.1. Ring Expansion Reactions for the Synthesis of Lactams

**Scheme 1:**
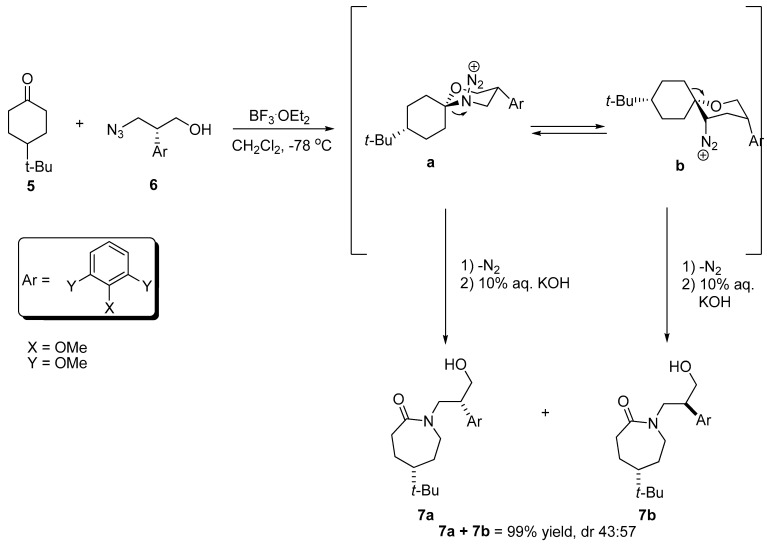
Reaction of hydroxyalkyl azide **6** and cyclic ketone **5** for the formation of substituted lactams **7a** and **7b** via intermediates **a** & **b**.

The effect of nonbonded, attractive cation–π interactions on the reactions of hydroxyl alkyl azides and cyclic ketones has been explored via computational and experimental sources. An attractive asymmetric pathway to lactams from the hydroxyalkyl azide-mediated ring expansion reaction of cyclic ketones was reported by Katz et al. The reaction of 2-aryl-1,3-hydroxyalkyl azide **6** with 4-*tert*-butylcyclohexanone **5** afforded the diastereomers of lactams **7a** and **7b** in maximum yields (99%) with 43:57 diastereomeric ratio. The solvent study revealed that out of different solvents such as toluene, diglyme, CH_2_Cl_2_, (C_5_H_5_)_2_O and *n*-C_5_H_12_, CH_2_Cl_2_ provided a 99% yield. The reaction proceeded well via asymmetric ring expansion reaction using BF_3_**^.^**OEt_2_, followed by the hydrolysis of intermediate iminium ethers using aqueous potassium hydroxide (Scheme 1) [20].

**Scheme 2:**
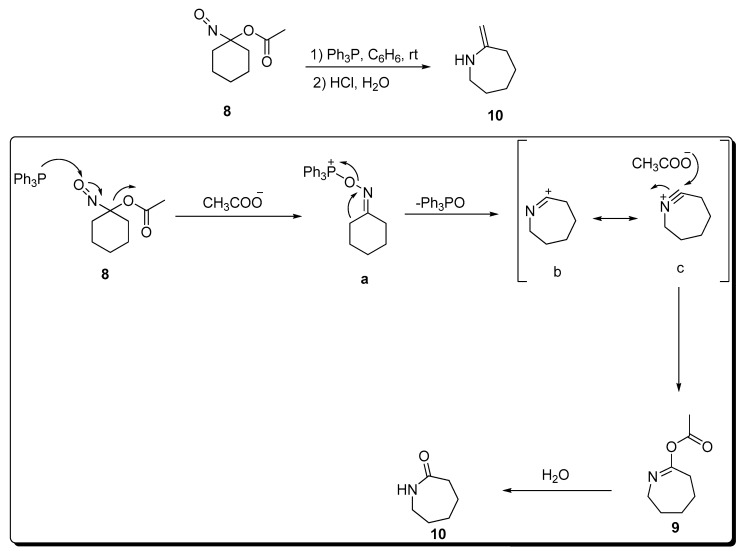
Formation of caprolactam **10** via ring expansion of acyloxy nitroso compound **8** via intermediates **a**, **b** and **c**.

Beckmann rearrangement is the rearrangement of an oxime functional group to an amide, which usually leads to ring expansion. Hadimani et al. reported the formation of lactam by ring expansion of acyloxy nitroso compound. 1-Nitrosocyclohexyl acetate **8** was treated with triphenylphosphine (TPP) in the presence of benzene, which gave seven-membered Beckmann rearrangement ring **9** in a 55% yield, along with triphenylphosphine oxide. Consequently, the intermediate **9** underwent acid-catalyzed hydrolysis (HCl 1M) to produce caprolactam **10** (Scheme 2) [16].

**Scheme 3:**
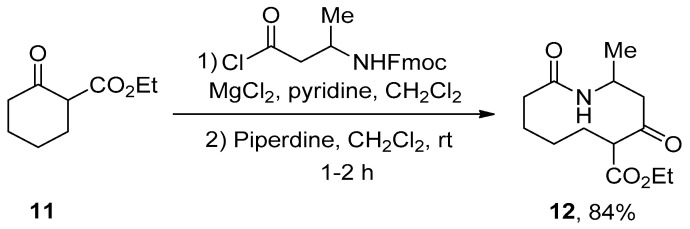
Formation of medium-sized lactam **12** via ring expansion reaction of *β*-keto ester **11**.

Medium and macrocyclic ring scaffolds have contributed significantly to medicinal chemistry [21]. Unsworth and his colleagues developed the synthetic approach for a wide range of medium-size lactams possessing medicinal lead-like properties. The lead-likeness of the medium-size lactams is analyzed through lead-likeness and molecular analysis (LLAMA). The 10-membered ring lactam **12** was obtained in an 84% (maximum) yield by ring expansion of *β*-keto ester **11** with acid chloride in the presence of MgCl_2_, pyiridine and dichloromethane, followed by the addition of piperidine and CH_2_Cl_2_. Room temperature was maintained for 1–2 h to carry out the reaction (Scheme 3). After achieving satisfactory results, authors applied this protocol on different substrates, affording substituted lactams in a 12–84% yield. This methodology demonstrates the worth of ring expansion reactions for synthesizing the medicinally appropriate compounds [22].

**Scheme 4:**
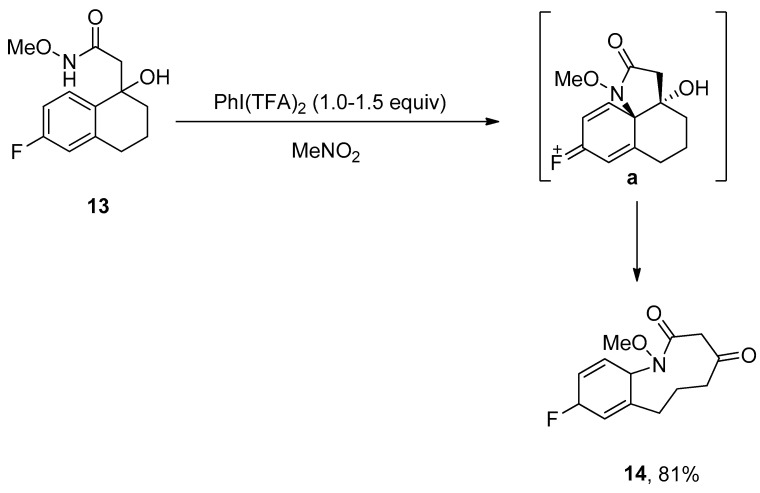
Synthesis of benzannulated medium ring lactam **14** via ODRE.

The effective and flexible protocol for the generation of benzannulated medium ring lactams from bicyclic scaffolds through oxidative dearomatization ring-expanding rearomatization reaction (ODRE) was outlined by Guney et al. A wide range of benzannulated lactams was attained in a 53–89% yield range by maintaining temperatures at 0–24 °C. However, the excellent yield (89%) of benzannulated lactam **14** was achieved by the reaction of chromanone-derived compound **13** with bis(trifluoroacetoxy)iodobenzene (PhI(TFA)_2_) in nitromethane as solvent for 0.5–2 h (Scheme 4). In contrast, the halobenzocyclooctanols and halobenzosuberanols afforded 10- and 11-membered benzannulated lactams in 70–90% yield [23].

**Scheme 5:**
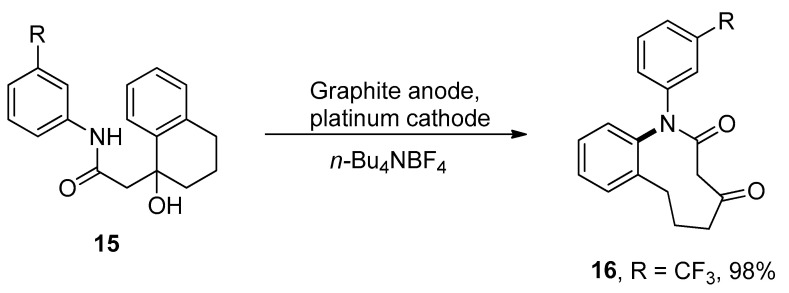
Formation of annulated medium-sized lactam **16** via electrochemical ring expansion reaction.

Xu et al. scrutinized the catalyst-free electrochemical ring expansion reaction for synthesizing the synthetically challenging annulated medium-size lactams via carbon–carbon bond cleavage. Highly functionalized medium-size lactam **16** was obtained in a 98% yield when CF_3_-aryl-substituted substrate **15** was electrolyzed in the electrolytic solution of *n*-Bu_4_NBF_4_ in CH_3_CN/H_2_O without using any catalysts or bases at 25 °C (Scheme 5). The electrolytic reaction shows compatibility with the electron-rich and electron-poor groups at either para- or metapositions of aniline to obtain a wide range of products (30–98%) [24].

**Scheme 6:**
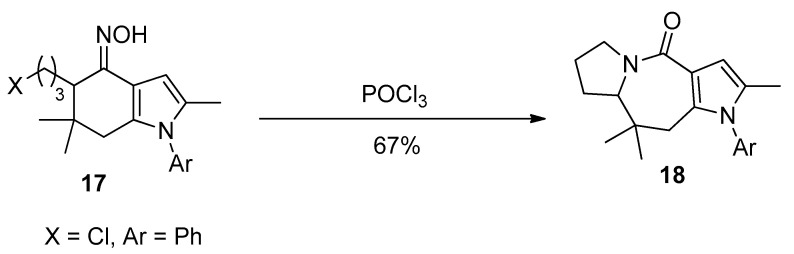
Formation of new fused polyheterocyclic lactam **18** via two-step tandem reactions.

The compounds containing the benzazepines motif have exhibited considerable importance in medicinal chemistry. For example, benazepril [2] is an angiotensin-converting enzyme inhibitor utilized to cure heart failure, hypertension [3] and diabetic kidney disease. Paullones [4] are the derivatives of indole [3,2-d]-1-benzoazepin-2-one that inhibit cyclin-dependent kinases (CDKs). Zarraga and coworkers performed the reaction of oxime **17** with POCl_3_ in order to obtain polyheterocyclic lactam. The reaction mixture was refluxed at 70 °C in dry THF for 1.5 h, giving a 67% yield of polyheterocyclic lactam **18**. The corresponding lactam was achieved by a tandem process involving Beckmann rearrangement/expansion and subsequent cyclization of nitrogen atom and primary halide fragment of **17** (Scheme 6) [5].

### 2.2. Ring Expansion Reactions for the Synthesis of Lactones

**Scheme 7:**
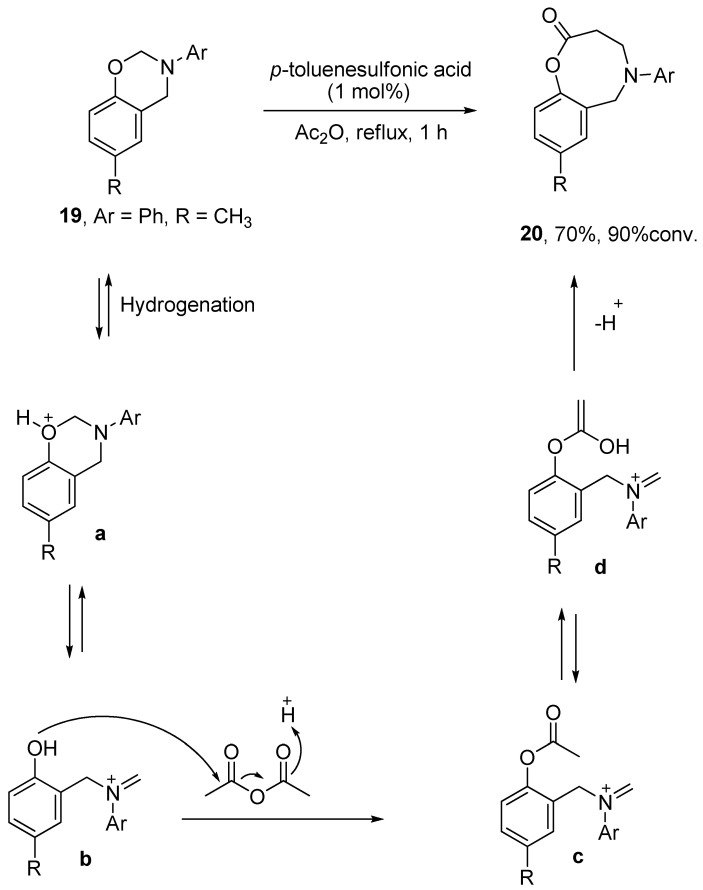
Ring expansion of benzoxazine derivative **19** for the formation of lactone **20**.

1,3-Benzoxazine, a class of cyclic compounds, has obtained appreciable attention because of its ring-opening polymerization that synthesizes polymers with excellent characteristics, such as thermal stability [25], high mechanical strength and durability under humid environment [26]. Endo and coworkers outlined the synthesis of eight-membered ring lactone by using benzoxazine as starting material. The solution of 1,3-benzoxazine **19** in acetic anhydride was refluxed using *p*-toluenesulfonic acid (1 mol%) as catalyst. The reaction mixture underwent ring expansion to afford eight-membered ring lactone **20**, containing a tertiary amine group in the ring, in a maximum of 70% yield with 90% conversion in 1 h (Scheme 7). For this purpose, the temperature outside the container was maintained at 150 °C [27].

**Scheme 8:**
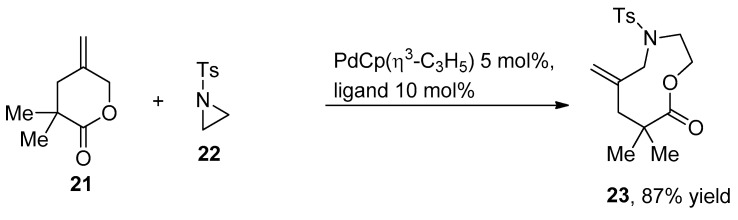
Ring expansion of valerolactone derivative **21** and aziridine **22** for the formation of azlactone **23**.

Shintani et al. in 2011 reported a ring expansion reaction by treating valerolactones **21** and aziridines **22** via (6 + 3) cyclization reaction, which resulted in the synthesis of nine atoms containing cyclic rings, referred to as “azlactones” **23** (Scheme 8). These azlactones are regarded as 1,4-oxanones, and they cannot be obtained simply by other reported methodologies in the past [28].

### 2.3. Ring Expansion Reactions for the Formation of Azulenes Derivatives

**Scheme 9:**
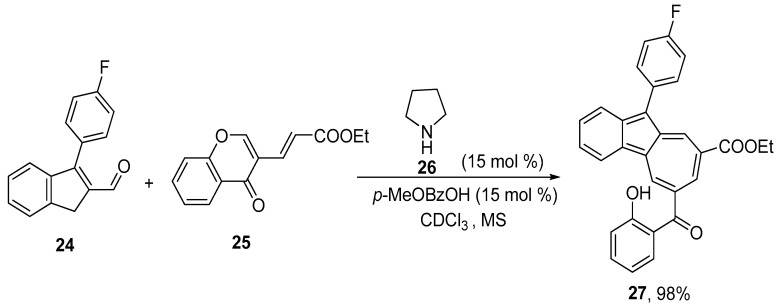
Synthesis of polysubstituted azulenes **27** via reaction of substituted indene 2-carbaldehyde **24** with chromen-4-one **25**.

Azulenes are composed of a five-membered ring fused to a seven-membered ring and are important colored compounds that are used for indicators [29], dyes [30] and imaging. Furthermore, these compounds show prominent biological and electronic properties. Gorgensen and colleagues synthesized a series of polysubstituted azulene derivatives through organocatalytic (10 + 4) cycloaddition of indene-2-carbaldehyde and chromen-4-one. The electron-deficient substituents provided azulenes in excellent yields (up to 98%), while electron-rich substituents gave different azulenes in the 45–83% yield range. The substituted azulene **27** was obtained in a 98% yield by reaction of substituted indene-2-carbaldehyde **24** with substituted chromen-4-one **25** in the presence of 15 mol% pyrrolidine **26**, 15 mol% *p*-MeOBzOH, CDCl_3_ and molecular sieves at 40 °C. (Scheme 9) [18].

### 2.4. Ring Expansion Reactions for the Synthesis of Azepine Derivatives

**Scheme 10:**
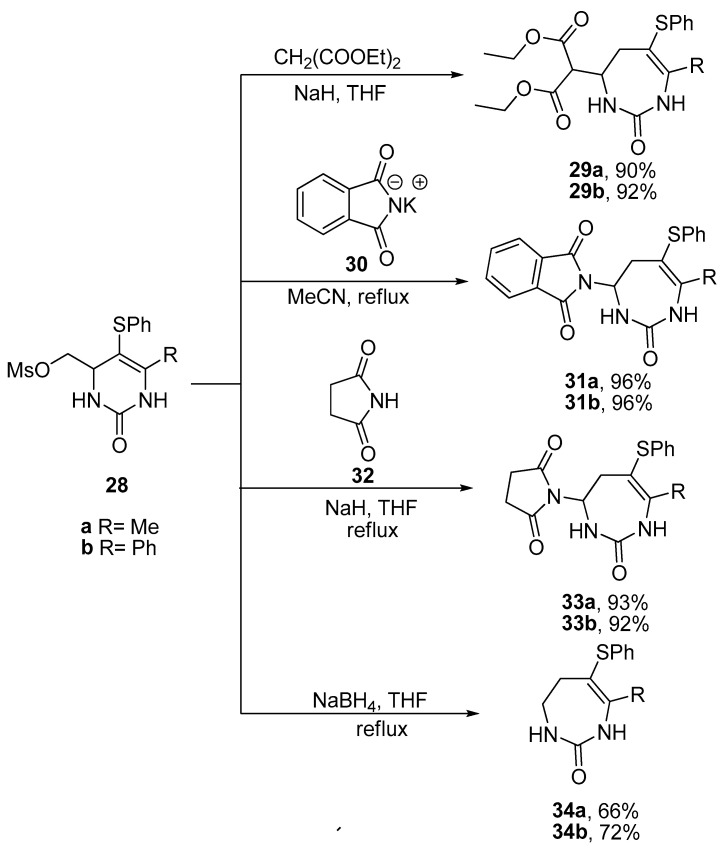
Formation of 1,3-diazepin-2-one derivatives **29(a,b), 31(a,b), 33(a,b) 34(a,b)** via ring expansion of pyrimidines **28a,b**.

The azepines class of heterocycles has gained tremendous importance due to wide-ranging biological activities. For example, some compounds are used to cure cardiovascular diseases and some are the inhibitors of cytidine deaminase [31]. A general approach towards the formation of 1,3-diazepin-2-one derivatives by the ring expansion reaction of pyrimidines using different nucleophiles was developed by Shutalev and coworkers. They synthesized different diazepinone derivatives in good-to-excellent yields under different reaction conditions. The reaction of **28a,b** (where R = CH_3_ & Ph respectively) and sodium diethyl malonate proceeded in THF at room temperature gives diazepinones **29a** and **29b** in 90% and 92% yields, respectively. 4-Mesyloxymethyl-pyrimidines **28a,b** reacted with potassium phthalimide **30** and refluxed in acetonitrile (MeCN) to afford the desired products **31a, b** in 96% yields. The pyrimidines **28a,b** refluxed in tetrahydrofuran in the presence of succinimide **32** and sodium hydride provided the diazepinones **33a** and **33b** in 93% and 92% yields, respectively. The 4-unsbstituted diazepinones **34a** and **34b** were attained in 66% and 72% yields, respectively, on refluxing **28a,b** with NaBH_4_ in THF. (Scheme 10) [32].

Fesenko et al. reported the nucleophilic-dependent diastereoselectivity of the ring expansion of pyrimidines with nucleophiles to synthesize polysubstituted1,3-diazepine. Several functionalized polysubstituted 1,3-diazepin-2-ones were obtained in excellent yields (80–97%) by the reaction of pyrimidines with different nucleophiles. The treatment of tetrahydropyrimidine **35** with MeONa in methanol gave a single cis-diastereomer **36** in a 93% yield. The reaction of **35** with EtONa in ethanol synthesized a diastereoselective (cis/trans = 93/7) 4-ethoxydiazepine **37** in a 97% yield.

**Scheme 11:**
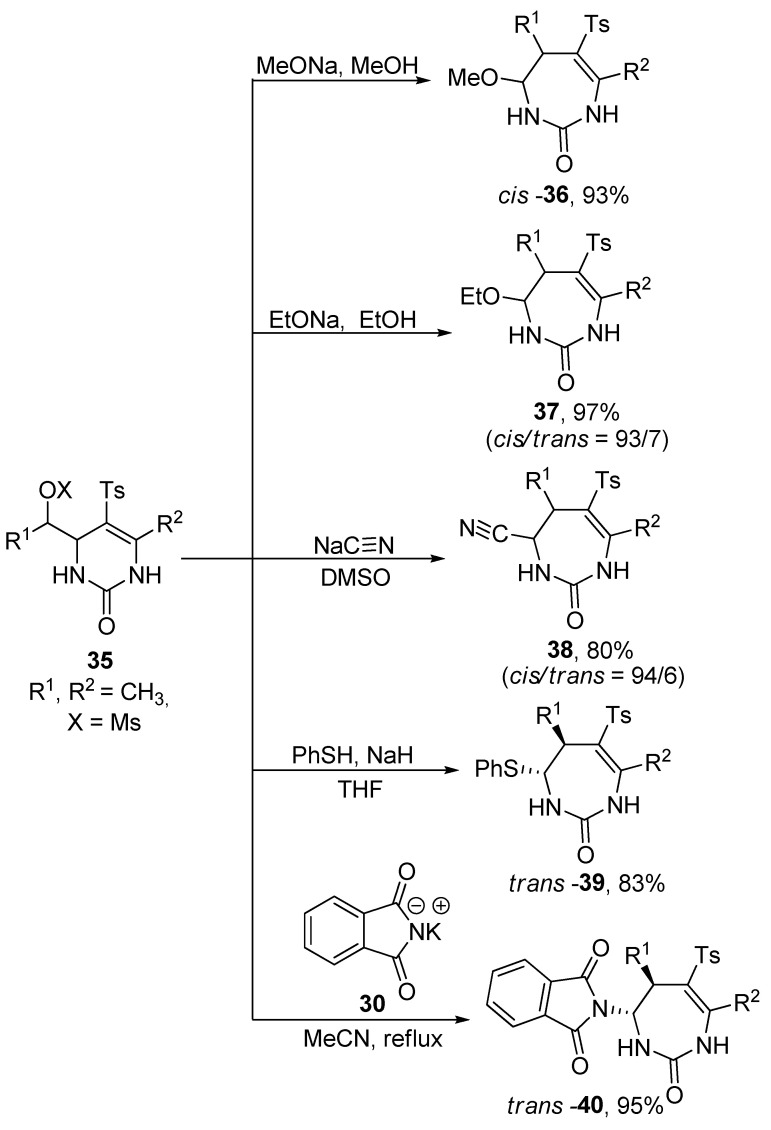
Synthesis of polysubstituted 1,3-diazepin-2-one **36, 37, 38, 39 and 40** by ring expansion reaction of pyrimidine **35** with nucleophiles.

When **35** was treated with sodium cyanide (NaCN) in DMSO at room temperature, a mixture of cis- and transdiastereomers (cis/trans = 94/6) **38** was obtained in an 80% yield. The transdiastereomer **39** was obtained in an 83% yield by the reaction of **35** with PhSNa, synthesized by the reaction of PhSH with sodium hydride in THF. The pyrimidine **35** and potassium phthalimide **30** were refluxed in acetonitrile, and the full trans-isomer **40** was obtained in a 95% yield (Scheme 11) [33].

**Scheme 12:**
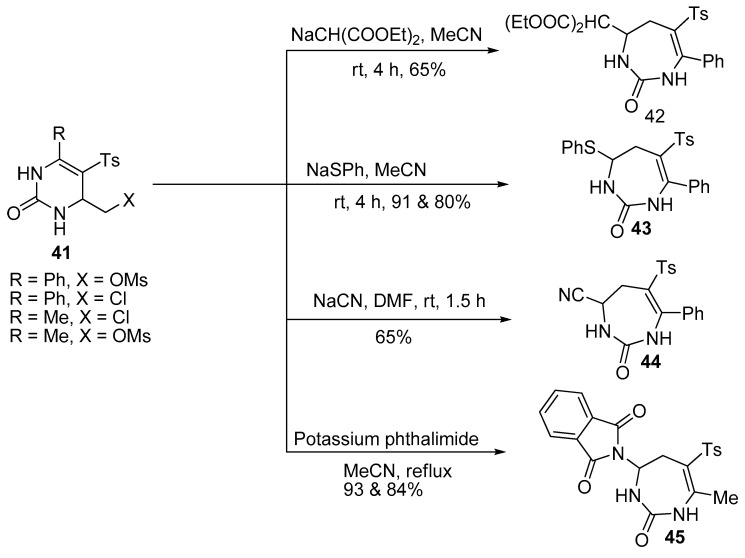
Synthesis of diversely substituted tetrahydro-1,3-diazepinone **42, 43, 44 and 45** as a result of ring expansion reaction of tetrahydropyrimidinones **41**.

Tetrahydrodiazepinones are essential biologically active heterocycles that have been rarely studied in the past due to the scarcity of their precursors. In order to obtain medicinally important diazepinones, Fesenko and coworker carried out the synthesis of diversely substituted 1,2,3,4-tetrahydropyrimidinones by reacting α-tosyl ketone enolates with *N*-[(2-benzoyloxy-1-tosyl)ethyl]urea. The synthesized tetrahydropyrimidinones **41** were then subjected to treatment with different nucleophiles, i.e., potassium phthalimide, sodium salt of diethyl malonate, sodium cyanide and sodium thiophenolate, to furnish tetrahydro-1,3-diazepinones **42, 43, 44** and **45** in good yields (Scheme 12) [34].

**Scheme 13:**
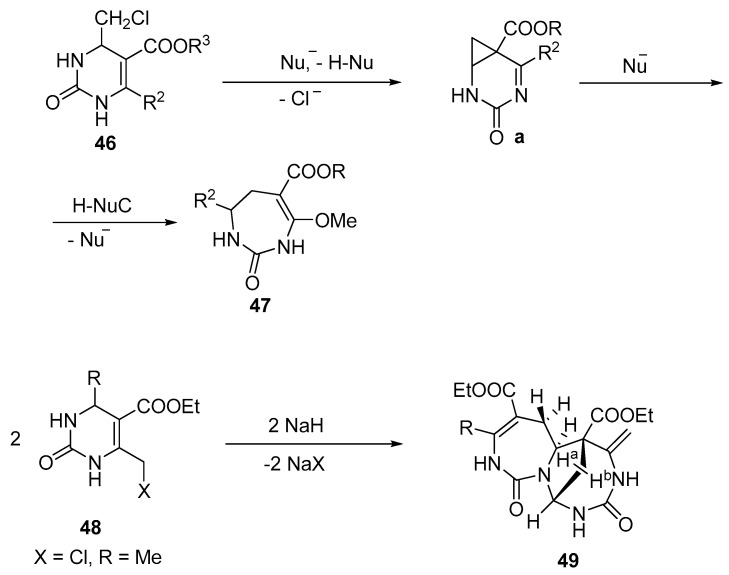
Synthesis of bis-diazepinone derivatives as a result of ring expansion of pyrimidinone derivatives.

Dihydropyrimidinones are easily accessible organic compounds; however, their extended seven-membered rings are rarely obtained. In order to obtain seven-membered cyclic system of Biginelli compounds, substituted Biginelli compounds **46** were treated with nucleophilic substances which first underwent removal of the proton, resulting in the generation of bicyclic intermediate as a result of subsequent nucleophilic substitution reaction. The intermediate then underwent ring expansion to give diazepinones **47** [35,36]. Shutalev et al., in 2008, employed easily accessible pyrimidinone derivative **48** to treat it with non-nucleophilic strong base, i.e., NaH, which furnished tricyclic bis-diazepinone derivative **49** as a result of tandem reaction (Scheme 13) [37].

**Scheme 14:**
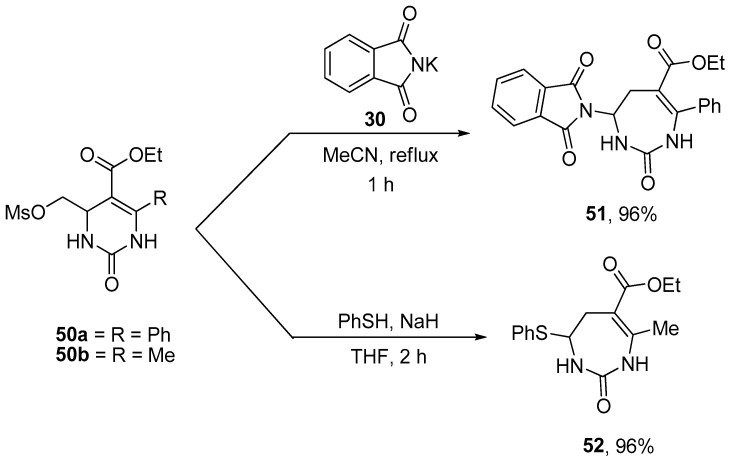
Synthesis of 1,3-diazepines **51** and **52** via ring expansion of acyl-substituted pyrimidine **50**.

Shutalev and coworkers outlined the synthetic protocol of diazepine by ring expansion of acyl-substituted pyrimidines. A series of diazepines was attained in a 43–96% yield range by applying different reaction conditions. The maximum (96%) yield of 1,3-diazepine **51** was obtained on refluxing 5-acyl-substituted pyrimidine **50a** with potassium phthalimide **30** in acetonitrile solvent for 1 h. Another diazepine derivative **52** was obtained in a 96% yield by the reaction of **50b** with thiophenol and sodium hydride in tetrahydrofuran (THF) for 2 h (Scheme 14). This protocol has a wide substrate scope, allowing the formation of substituted diazepines (80–96%) [38].

### 2.5. Ring Expansion Reaction for the Synthesis of Tropone Derivatives

**Scheme 15:**
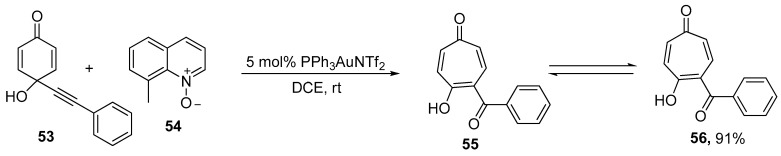
Synthesis of tropone derivatives **56** as a result of ring expansion reaction of alkynyl quinols.

Tropones have gained significant importance owing to their unique structural features, along with their remarkable biological activities. They are found to be essential structural motifs of various naturally occurring and medicinally important organic compounds. The ring elongation reaction of alkynyl quinols results in the synthesis of tropone derivatives. Considering the wide applications of tropone derivatives, Zhao et al., in 2015, reported the synthesis of tropone derivatives by employing the ring elongation reaction of alkynyl quinols in the presence of various gold catalysts and a diverse range of solvents. The optimization reactions revealed that the utilization of PPh_3_AuNTf catalyst and DCE solvent resulted in high yields of target molecules (Scheme 15) [39].

**Scheme 16:**
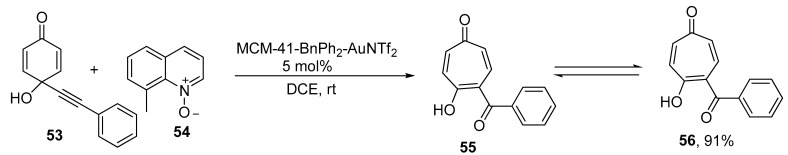
Synthesis of tropone derivatives **56** as a result of ring-expansion reaction of alkynyl quinols **53**.

In 2021, Du et al. reported the ring extension reaction of alkynyl quinols by employing a highly efficient catalyst, i.e., an MCM-41-based confined complex of gold carrying altered benzylidene phosphine. MCM-41-BnPh_2_-AuX is regarded as a productive catalyst owing to its large surface area, resistance to high temperature and facile recycling properties. Taking into account the usage of this effective catalyst, Du et al. synthesized and then incorporated this catalyst into the expansion reaction of alkynyl quinols. The reaction conditions were optimized by using various solvents and by varying X in gold complex catalyst. However, the highest yield (91%) was obtained by using DCE as solvent, along with the incorporation of NTf_2_ in MCM-41-BnPh_2_-AuX (Scheme 16) [40].

### 2.6. Miscellaneous Ring Expansion Reactions

**Scheme 17:**
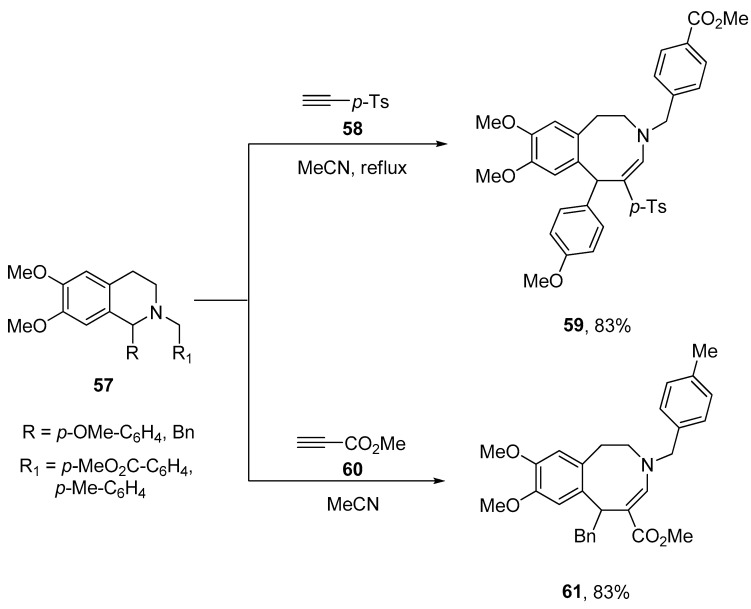
Synthesis of tetrahydrobenzoazocines **59** and **61** via ring expansion reaction of tetrahydroisoquinoline **57** with activated alkynes **58** and **60**.

Benzoazocine-containing drugs and alkaloids exhibit numerous biological activities, e.g., antitumor activity [10], antinociception activity [12], inhibition of *α*-glucosidase [14], acetylcholinesterase [41] and inhibition of HIV-1 replication [11]. Voskressensky and coworkers reported the synthesis of benzoazocines via the ring enlargement of substituted tetrahydroisoquinolines and activated alkynes. A total of 37–83% of substituted tetrahydrobenzoazocines was achieved by the reaction of different tetrahydroisoquinolines with alkynes using acetonitrile as solvent. The tetrahydroisoquinoline **57** was refluxed with alkyne **58** in acetonitrile, giving corresponding tetrahydrobenzoazocines **59** in an 83% yield. In addition, the maximum (83%) yield of **61** was obtained by the reaction of **57** and **60** (Scheme 17). Acetonitrile was selected as the appropriate solvent for this reaction [42].

**Scheme 18:**
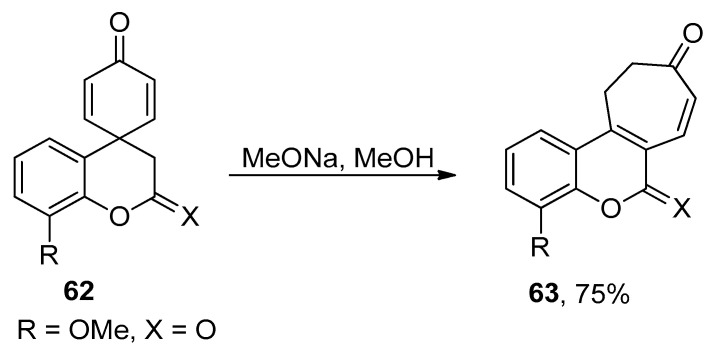
Synthesis of dihydrotropones **63** via rearrangement of spirocyclichexadienone **62**.

The seven-membered carbocyclic rings are mostly found in potent medicinal scaffolds such as thapsigargins [43], colchicines [44], hinokitiol [45], etc. The ring expansion reaction for the formation of dihydrotropones conducted by the rearrangement of spirocyclohexadienones was performed by Guillou and coworkers. To achieve the targeted dihydrotropone **63**, authors utilized spirocyclichexadienone **62** as the starting precursor. For this protocol, MeONa in methanol was selected as an effective reaction media. An excellent yield (75%) of dihydrotropone **63** was attained by the rearrangement of spirocyclichexadienone **62** within 30 min by keeping the temperature at 40 °C (Scheme 18) [46].

**Scheme 19:**
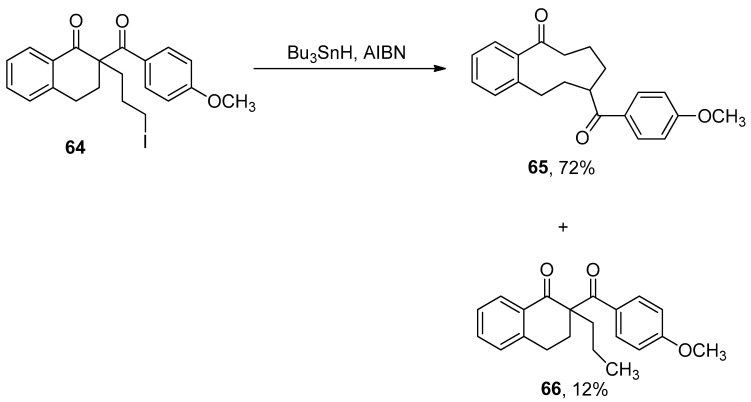
Synthesis of nine-membered 1,6-diketone **65/66** via three-carbon ring expansion and cyclization of 1,3-diketones **64**.

Free radical ring expansion reactions of five and six-membered rings have extended the use of radical chemistry for framing the large cyclic compounds. Xu et al. reported the ring expansion reaction promoted by free radical and associated cyclization of 1,3-diketone derivatives. A 68–72% yield range of targeted nine-membered ring compounds was attained under optimized reaction conditions (Bu_3_SnH, AIBN, C_6_H_6_ and 80 °C). The compound **56** was refluxed in benzene using tri-*n*-butyltin hydride (Bu_3_SnH) and azobisisobutyronitrile (AIBN) as catalyst. As a result, targeted derivative **57** was achieved as the major product, along with direct reduction product **58** in a 12% yield by maintaining the temperature at 80 °C (Scheme 19) [47].

**Scheme 20:**
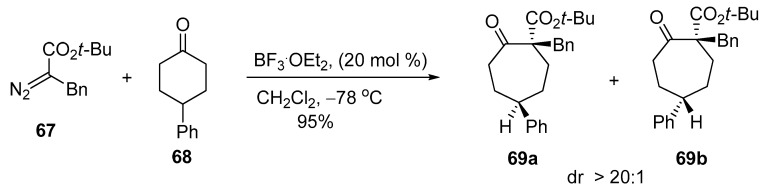
Formation of diastereoselective seven-membered ring carbocycles **69a** and **69b** via Tiffeneau–Demjanov-type ring expansion reaction.

Seven-membered carbocycles gained substantial attention due to their remarkable biological activities. Maruoka and colleagues made a novel contribution to the stereoselective construction of seven-membered ring compounds through direct Tiffeneau–Demjanov-type ring enlargement. The authors performed the reaction between *t*-butyl *α*-benzyl diazoacetate **67** and 4-phenylcyclohexanone **68**. Resultantly, the corresponding product with one all-carbon quaternary center was obtained in a 95% yield. To facilitate this approach, optimized reaction conditions involved 20 mol% BF_3_**^.^**OEt_2_ (catalyst), CH_2_Cl_2_ (solvent), 30 min and −78 °C temperature (Scheme 20). The 40–95% yield range proved the effectivity of this methodology [17].

**Scheme 21:**
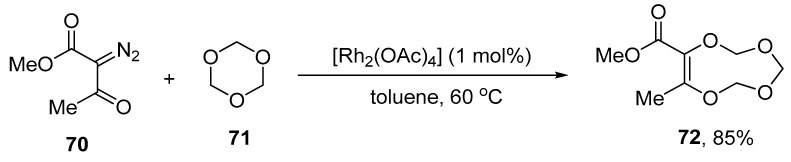
Rhodium-catalyzed ring expansion for synthesis of medium-sized ring compounds **72**.

Ballesteros-Garrido et al. reported the synthesis of medium-size rings by using rhodium-catalyzed ring expansion reaction in one pot. Several functionalized nine-membered ring compounds were isolated in the low-to-high yields (31–85%) by the reaction of *α*-diazodicarbonyls with cyclic acetals. *α*-diazo *β*-ketoester **70** underwent reaction with 1,3,5-trioxane **71** at 60 °C using (Rh_2_(OAc)_4_) (1 mol%) as catalyst (Scheme 21). Toluene proved to be a suitable solvent for this reaction. Consequently, an 85% yield of corresponding product **72** was achieved as a single product [48].

**Scheme 22:**
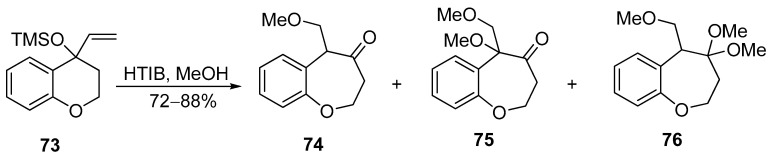
Formation of seven-membered rings **74**, **75** and **76** by ring expansion of 1-vinylcycloalkaol derivative **73**.

Seven-membered ring compounds possessing enormous biological activities have attracted the attention of chemists to develop synthetic routes for their preparation. Silva et al. reported the metal-free, versatile and efficient method for synthesizing hetero- and carbocycles containing seven-membered rings. 1-Vinylcycloalkanol derivative **73** underwent hydroxy(tosyloxy)iodobenzene (HTIB)-promoted ring expansion. As a result, a mixture of O-heterocycles **74**, **75** and **76** was attained in a 72–88% yield range (Scheme 22). The reaction parameters for the corresponding reaction include HTIB, a hypervalent iodine reagent and methanol as solvent. The easy availability of starting materials is the salient feature of this protocol [49].

**Scheme 23:**
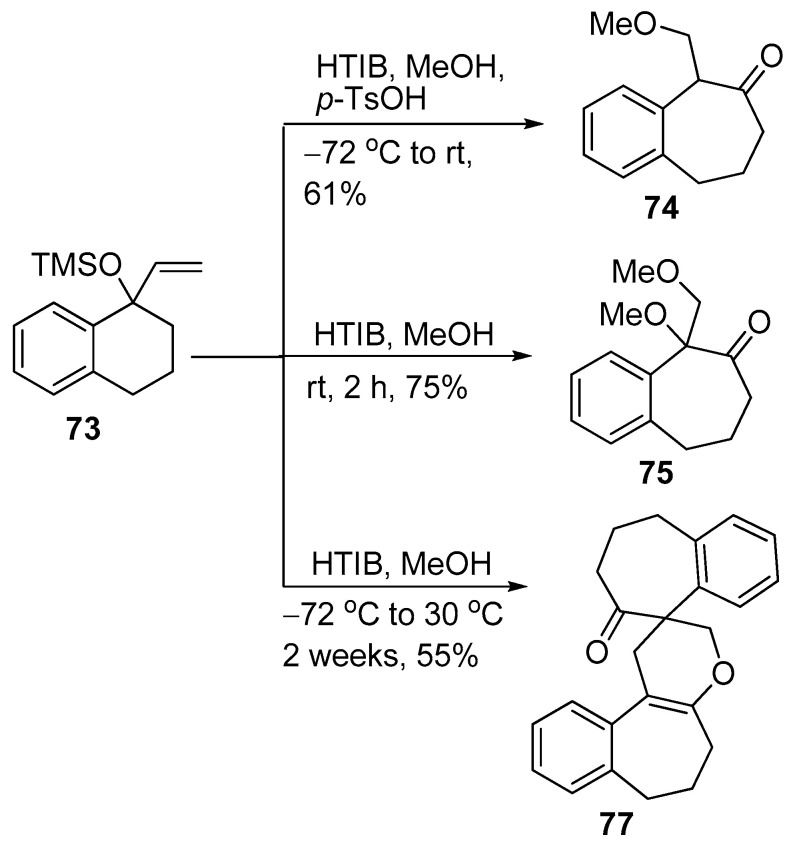
Formation of seven-membered rings **74, 75 and 77** by ring expansion of 1-vinylcycloalkaol derivatives **73**.

Seven-membered heterocycles are core structures of numerous naturally occurring biologically potent organic compounds. Cancer is the most prevent and deadly disease in the recent era, and continuous efforts are in progress to devise anticancerous agents [50]. For example, theaflavin is known to inhibit the proliferation of cancerous cells [51]. Similarly, TAK-779 is highly effective against human immunodeficiency virus [52]. There have been numerous reports concerning the synthesis of seven-membered cyclic organic compounds via utilization of titanium, palladium or mercury metals. Silva et al., in 2008, carried out the ring expansion reaction of six-membered cyclic rings by devising a metal-free synthetic approach. For this purpose, they treated silyl or methyl ether of vinylcycloalkanols in the presence of HTIB (PhI(OH)OTs) and methanol to furnish lactone-based cyclic compounds **77**. Similarly, the reaction to silyl enol ether of vinylcycloalkanols **73** with iodine’s reagent by using methanol as solvent in the presence of *p*-TsOH led to the synthesis of methoxy ketone **74** in a 61% yield. However, the reaction of vinylcycloalkanol with 2.5 equivqlents of HTIB in the presence of methanol achieved dimethoxyketone **75** in a 75% yield (Scheme 23) [53].

**Scheme 24:**
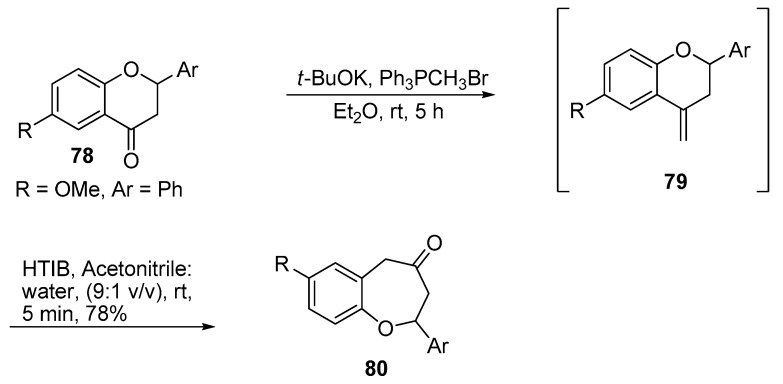
Formation of seven-membered rings **80** by ring expansion of 1-vinylcycloalkaol derivatives **78**.

Oxygen, sulfur and nitrogen constituting heterocycles are diversely and abundantly found in various pharmaceutically important organic compounds. For example, bauhiniastatin is a naturally occurring seven-membered cyclic ring that has been found to be a potent cytotoxic agent. Taking into account the unparalleled pharmacological applications of seven-membered cyclic rings, Khan et al. in 2021 attempted their synthesis via ring expansion reaction by utilizing an iodine reagent, i.e., HTIB (PhI(OH)OTs), as catalyst. HTIB (PhI(OH)OTs) is regarded as Koser’s reagent and has been found to be highly effective as an alternative to expensive metal catalysts that were previously employed for the synthesis of seven-membered heterocyclic rings. Khan et al. reacted ketone **78** with potassium *tert*-butoxide, Ph_3_PCH_3_Br and diethyl ether, which resulted in the synthesis of chromane intermediate **79**. The synthesized chromane was then treated with HTIB catalyst in the presence of acetonitrile and water as solvent to synthesize seven-membered heterocycle **80** in a good yield (Scheme 24) [54].

**Scheme 25:**
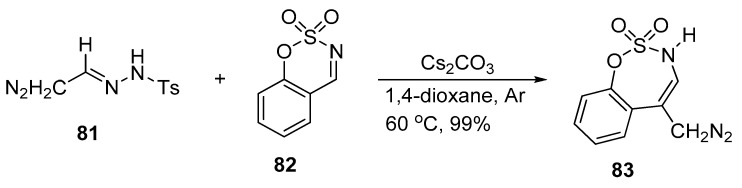
Synthesis of seven-membered enesulfonamide **83** under argon atmosphere.

Seven-membered sulfonamides possess enormous biological activities, such as the inhibition of HIV-1 protease [55] calcium-sensing receptor agonists [56] and apical sodium-dependent bile acid transporter (ASBT) inhibition [57]. Moreover, they act as synthetic intermediates for synthesizing biologically active scaffolds. Xia et al. reported the reactions of *N*-sulfonylimines with diazomethane under metal-free conditions providing enesulfonamides in a 45–99% yield range. The reaction worked efficiently by using Cs_2_CO_3_ as base and 1,4-dioxane at 60 °C. A maximum (99%) yield of enesulfonamide **83** was obtained under argon atmosphere (Scheme 25) [58].

**Scheme 26:**
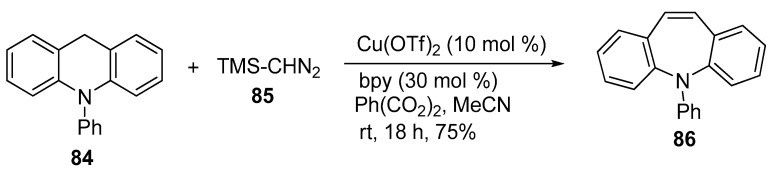
Copper-catalyzed ring expansion for the formation of tricyclic dibenzoxepines and dibenzazepines **86**.

Tricyclic dibenzazepines and dibenzoxepines derivatives are therapeutic agents and possess a wide range of pharmaceutical properties, for example, metapramine [59] and opipramol [9] display analgesic and anxiolytic properties, respectively. In addition, oxcarbazepine, an antiepileptic drug (AED) [13], is used as monotherapy for medication of partial seizures in adults. Mancheno and coworkers reported the facile and effortless way to synthesize dibenzazepines and dibenzoxepines oxidative C-H bond rearrangement and ring expansion using copper catalyst. The tricyclic product **86** was obtained in a 75% yield by the reaction of **84** with TMS-CHN_2_ **85** in acetonitrile. The reaction was completed in 18 h using 10 mol% Cu(OTf)_2_ as catalyst, 30 mol% of 2,2-bipyridine as ligand and Ph(CO_2_)_2_ as additive (Scheme 26). Electron-donating (OMe, Ph, Me) and electron-withdrawing (F, Br) substituents were well-tolerated in the corresponding reaction, giving 55–74% yields of respective products. The substitution of O by N-Ph group gave a 55% yield, while the substitution of sulfur gave a 36% yield of the corresponding product [8].

**Scheme 27:**
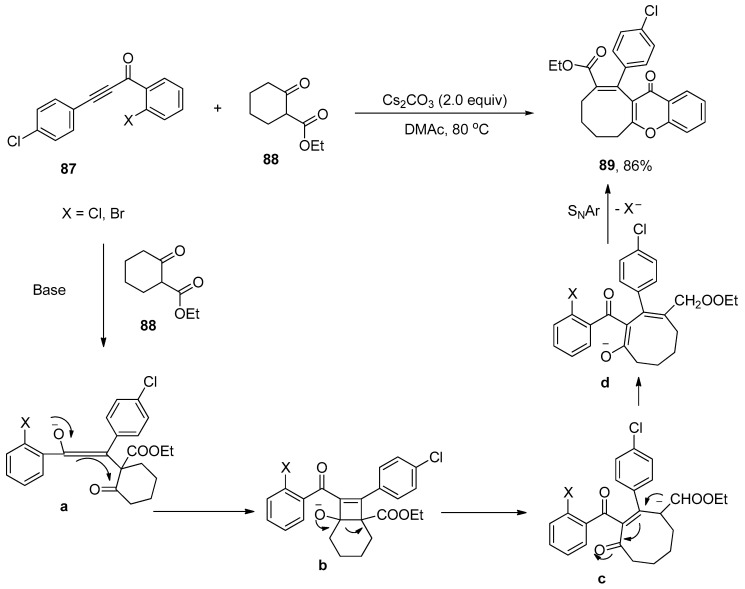
Formation of eight-membered fused ring **89** by insertion of alkynes **87** into *β*-diketone **88**.

The insertion of isolated alkynes into the carbon–carbon σ-bond of unstrained cyclic *β*-dicarbonyl compounds without use of transition metals is outlined by Zhou et al. The reaction of different alkynes and dicarbonyl compounds consequently gave corresponding ring expansion products in a 49–86% yield range. It was noted that electron-donating and -withdrawing substituents on aryl groups of alkynes gave respective products in good yields. The insertion of alkynes **87** into cyclic *β*-diketone **88** was facilitated under optimized reaction conditions (Cs_2_CO_3_, DMAc, 80 °C). The 2.0 equiv cesium carbonate (Cs_2_CO_3_) was used as base and dimethylacetamide (DMAc) as an effective solvent, as compared with DMF, DMSO and toluene, to attain the required product **89** in an 86% yield (Scheme 27). Due to mild reaction conditions and easily accessible starting precursors, this approach is applicable to organic synthesis [60].

**Scheme 28:**
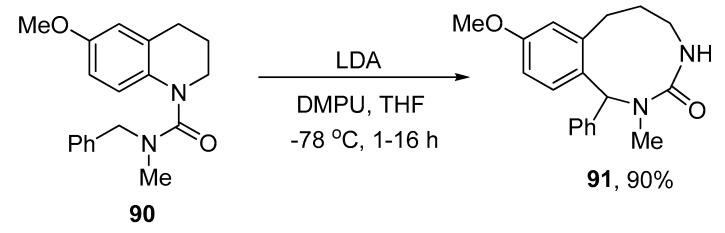
Synthesis of medium-sized benzodiazonine **91** via migratory ring expansion of urea derivatives **90**.

The existence of 8- to 12-membered medium-ring heterocycles in natural products demonstrates several biological activities. Clayden and coworkers reported an effective method to synthesize medium (8- to 12-membered) benzo-fused nitrogen-containing heterocyclic rings via n → n + 3 ring expansion of metalated urea. A series of substituted nine-membered benzodiazonines in a 50–90% yield proved the substrate scope and efficacy of this protocol. The reaction was processed in tetrahydrofuran in the presence of lithium diisopropylamide (LDA) as base and *N*,*N*^’^-dimethylpropylideneurea (DMPU) as additive. By maintaining temperature at −78 °C, product **91** was attained through migratory ring expansion reaction of urea derivative in 1–16 h. The investigation of bases such as LDA and sec-BuLi was conducted. LDA was selected as a suitable base for this reaction (Scheme 28) [61].

**Scheme 29:**
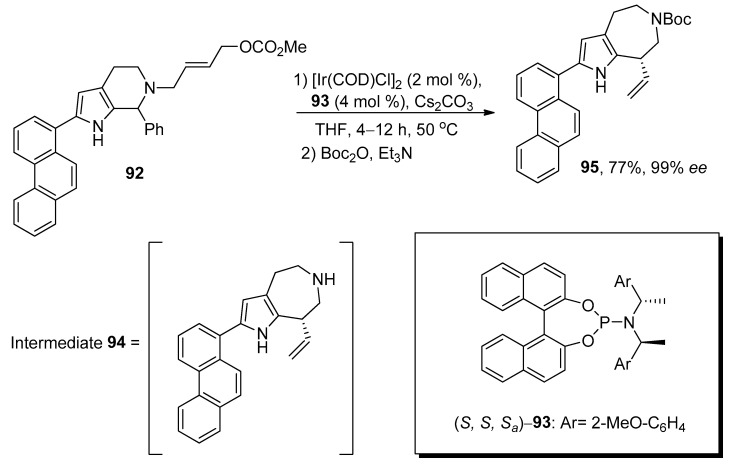
Formation of pyrrole-annulated medium-sized rings **95**.

The pyrrole-annulated medium-sized rings have attracted considerable attention, as they are found in pharmaceutical agents, naturally occurring products and biologically active compounds. For example, some of the compounds show useful effects on the central nervous system and some are agonists of the farnesoid X receptor [62]. The enantioselective construction of pyrrole-annulated seven-membered rings by allylic dearomatization or retro-Mannich/hydrolysis via iridium catalyst was reported by Huang et al. The targeted product **95** was obtained in a 77% yield and an enantioselectivity value of 99% ee by the reaction of 92 with 2 mol% of iridium catalyst (Ir(COD)Cl)_2_ and 4 mol% of ligand **93**. The reaction worked well by utilizing 100 mol% (Cs_2_CO_3_) as base in THF through an intermediate **94**, followed by the Boc protection of amino group by using Boc_2_O and triethylamine (Et_3_N) (Scheme 29). The reaction was completed in 4–12 h by keeping the temperature at 50 °C [63].

**Scheme 30:**
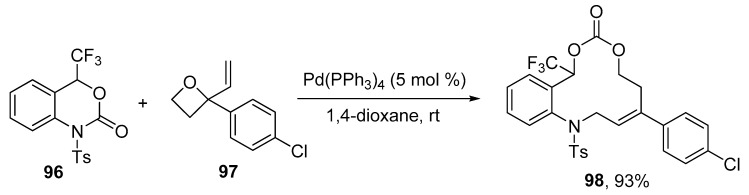
Synthesis of 12-membered heterocycles **98** via decarboxylative Pd-catalyzed (6 + 6) annulation.

Oxetanes are abundantly present in several medicinal drugs [64]. Shibata and coworkers reported a simple method for the formation of 12-membered trifluoromethyl heterocycles through nondecarboxylative palladium-catalyzed (6 + 6) annulation of 6-membered trifluoromethylbenzo[d][1,3]oxazinones and several vinyl oxetanes. Vinyl oxetanes containing electron-rich (4-OCH_3_, 2-OMe, 4-CH_3_) and electron-poor (4-Cl, 4-Br, 4-F) substituents on aryl ring gave respective products in good-to-excellent yields (66–93%). The impact of different solvents (THF, CH_2_Cl_2_, 1,4-dioxane, MeCN and toluene) was examined, and results declare that 1,4-dioxane gave the maximum yield, as compared with other solvents. Highly functionalized trifluoromethyl 12-membered ring heterocycle **98** was obtained in an excellent yield (93%) by the reaction of **96** with substituted vinyl oxetane **97** using 5 mol% Pd(PPh_3_)_4_ via Pd-catalyzed non-decarboxylative (6 + 6) annulation (Scheme 30) [19].

**Scheme 31:**
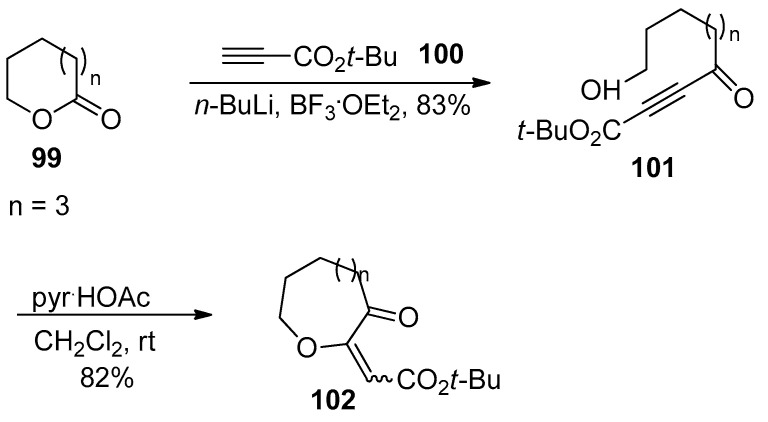
Ring expansion reaction of lactones or lactams **99**.

Most of the pharmaceutically important organic compounds contain saturated cyclic rings having nitrogen and oxygen atoms. The synthesis of heterocycles containing more carbon atoms is a very interesting task, which can be easily accomplished by employing the ring closing strategy on open-chain starting materials. However, Grant et al., in 2008, reported the synthesis of these heterocycles by subjecting lactams or lactones to the ring-expansion reaction. For this purpose, they treated *N*-sulfonyllactams **99** and easily available lactones with *tert*-butyl propiolate **100** in the presence of *n*-butyl lithium and boron triflouride diethyl etherate, which gave acyclic adducts **101** in good yields. The synthesized adducts were then made to react with pyridinium acetate, which resulted in the synthesis of six or seven carbon-containing cyclized ethers or amines upon cyclization **102** (Scheme 31). The higher carbon-containing cyclic rings are synthesized as a result of ring elongation methodology, which proceeds via nucleophile-catalyzed reaction [65].

**Scheme 32:**
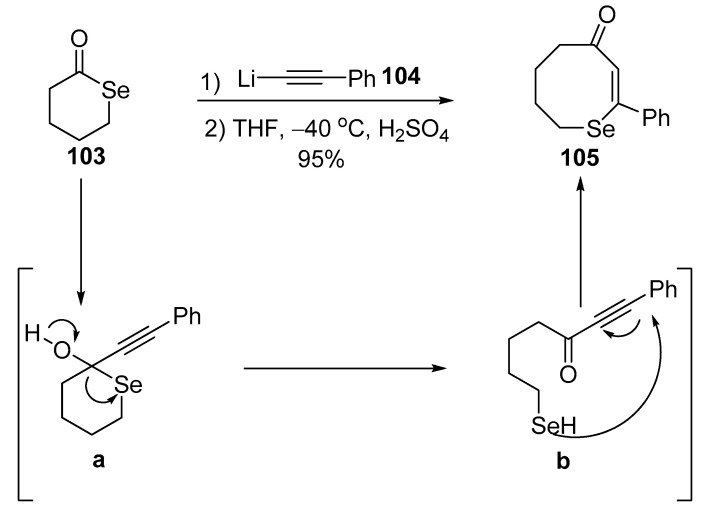
Ring expansion reaction between tertrahydroselenopyranone **103** and diversely substituted ethynyl lithiums **104** for formation of eight-membered heterocyclic ring **105**.

Selenium-incorporated heterocycles have gained considerable significance due to their significant biological applications and extraordinary chemical properties [66]. However, there are few reports concerning the synthesis of selenium-based cyclic rings. Thus, in 2008, Sashida et al. reacted tertrahydroselenopyranone **103** with diversely substituted ethynyl lithiums to synthesize selenium-incorporated eight-membered heterocyclic ring **105**. The reaction was carried out under different conditions, and the highest yield (95%) was obtained when the reaction between the two substrates was carried out by substituting R = Ph and by employing THF as solvent at −40 °C in the presence of sulfuric acid as proton source (Scheme 32) [67].

**Scheme 33:**
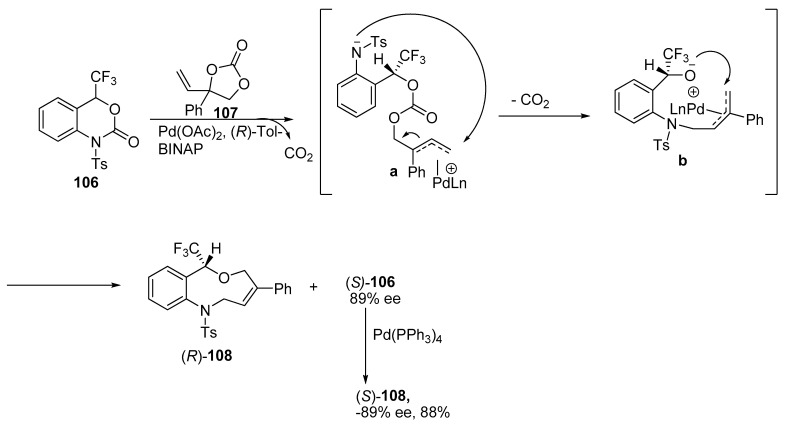
Ring expansion reaction of [1,3]-oxazinones **106** and vinyl-substituted ethylene carbonates **107** to synthesize [1,5]-oxazinone derivatives **108**.

A wide variety of medicinal drugs constitute fluorine constituting organic compounds, which emphasizes the synthesis of fluorine-based organic molecules. According to the reported data by the FDA in 2018, almost 47% percent of approved drugs include fluorine/CF_3_ in their structural formula [68]. Considering the wide applicability of fluorine-based organic molecules, Uno et al. carried out the ring extension reaction of trifluoromethyl-benzo [1,3]-oxazinones by employing an optically active ligand as catalyst. In this regard, they reacted [1,3]-oxazinones with vinyl-substituted ethylene carbonates in the presence of palladium acetate and (*R*)-Tol-BINAP, which led to the synthesis of a nine-membered heterocycle, i.e., dextrorotary and levorotatory mirror images of trifluoromethyl-substituted [1,5]-oxazonines in high enantiomeric excess (up to 98% ee). In the first step, double removal of carboxylic acid results in ring elongation to furnish phenyl sunstituted *(R*)-**108**. The nonreactive starting material in the second cycle is then converted to (*S)-***108** in 88% yield with -89%ee without utilizing another optically active ligand. (Scheme 33). However, thiophene substituted ethylene carbonate resulted in -98% ee of target molecule in 81% yield [69].

## 3. Conclusions

In summary, the synthesis of biologically active carbocyclic/heterocyclic compounds has been achieved by numerous pathways. The current review article outlines a wide range of reactions to demonstrate the synthetic importance of functionalized carbocyclic/heterocyclic molecules formed via six-membered ring expansion reactions that covers the literature reported from 2017 to 2022. The use of catalysts and ligands and the effect of different substituents on product yields while conducting reactions were also illustrated in this review. Moreover, such ring expansion reactions will provide meticulous guidelines for synthetic and pharmaceutical chemists to yield valuable ring expansion products.

## Data Availability

Data are available in the manuscript.
